# Endoglin and Systemic Sclerosis: A PRISMA-driven systematic review

**DOI:** 10.3389/fmed.2022.964526

**Published:** 2022-08-18

**Authors:** Silvia Grignaschi, Anna Sbalchiero, Giuseppe Spinozzi, Bianca Lucia Palermo, Claudia Cantarini, Chantal Nardiello, Lorenzo Cavagna, Carla Olivieri

**Affiliations:** ^1^Department of Internal Medicine and Medical Therapeutics, University of Pavia, Pavia, Italy; ^2^Rheumatology Division, Fondazione Istituti di Ricovero e Cura a Carattere Scientifico (IRCCS) Policlinico San Matteo, Pavia, Italy; ^3^General Biology and Medical Genetics Unit, Department of Molecular Medicine, University of Pavia, Pavia, Italy; ^4^Otorhinolaryngology Division, Fondazione Istituti di Ricovero e Cura a Carattere Scientifico (IRCCS) Policlinico San Matteo, Pavia, Italy

**Keywords:** systemic sclerosis, Endoglin, sENG, TGFβ pathway, PRISMA review

## Abstract

**Background:**

Systemic Sclerosis (SSc) is a rare autoimmune disease whose pathogenesis is still poorly understood. The Transforming Growth Factor β superfamily is considered pivotal and a crucial role has been suggested for the type III receptor, Endoglin (ENG). The aim of this systematic review is to investigate and combine the current clinical and molecular available data, to suggest novel hints for further studies.

**Methods:**

We followed PRISMA guidelines; the search was performed on three databases (MEDLINE, Web of Science, Embase) in date November 2nd, 2021. Subsequent to the exclusion of duplicates, we applied as inclusion criteria: 1. focus on the relationship between ENG and SSc; 2. English language. As exclusion criteria: 1. ENG exclusively as a cellular biomarker; 2. no focus on ENG-SSc relationship; 3. review articles and 4. abstracts that did not add novel data. Eligibility was assessed independently by each author to reduce biases. We divided records into clinical and molecular works and subgrouped them by their study features and aim.

**Results:**

We selected 25 original papers and 10 conference abstracts. Molecular studies included 6 articles and 4 abstracts, whereas clinical studies included 17 articles and 6 abstracts; 2 articles presented both characteristics. Molecular studies were focussed on ENG expression in different cell types, showing an altered ENG expression in SSc-affected cells. Clinical studies mainly suggested that different disease phenotypes can be related to peculiar disregulations in soluble ENG concentrations.

**Discussion:**

Concerning the possible limits of our search, boolean operators in our strings might have been uneffective. However, the use of different strings in different databases should have reduced this issue at a minimum. Another bias can be represented by the selection step, in which we excluded many articles based on the role of Endoglin as a histological vascular marker rather than a signaling receptor. We tried to reduce this risk by performing the selection independently by each author and discussing disagreements. Our systematic review pointed out that ENG has a pivotal role in activating different TGFβ-stimulated pathways that can be crucial in SSc pathogenesis and progression.

## Introduction

### Clinical overview of systemic sclerosis

Systemic Sclerosis (SSc) is a rare autoimmune disease which affects connective tissue with widespread vasculopathy and inflammation, leading to an excess of collagen fibers deposition in the skin and internal organs. Skin thickening is the hallmark of the “scleroderma spectrum disorders,” a group of clinical entities which encompasses different manifestations ranging from localized forms only confined to the skin, such as morphea or localized scleroderma (LoS), to systemic forms with progressive cutaneous fibrosis and involvement of internal tissues (1). Based on the extent of skin thickening, patients are generally classified into diffuse cutaneous systemic sclerosis (dcSSc) or limited cutaneous systemic sclerosis (lcSSc), the latter being previously known as “CREST” syndrome (calcinosis, Raynaud's Phenomenon, esophageal dysmotility, sclerodactyly, telangiectases). A small subset of patients shows clinical features and autoantibodies specific to SSc in the absence of skin hardening (SSc *sine scleroderma*) (2).

SSc carries high mortality risks related to disease complications and brings along significant morbidities with negative impact on quality of life and function. Over the last few years, a growing interest in the very early diagnosis has led to the identification of “red flags”: puffy fingers, Raynaud's Phenomenon (RP) and positive antinuclear antibodies (ANA), along with the presence of capillaroscopic abnormalities and/or disease-specific autoantibodies (3). The autoimmune profile is complementary to the cutaneous subtype in defining the wide spectrum of SSc clinical features and it contributes to the prognostic stratification. Anticentromere antibodies (ACA) are generally associated with lcSSc, calcinosis, telangiectases and pulmonary arterial hypertension (PAH); anti-topoisomerase I antibodies are related to dcSSc, progressive lung fibrosis and digital ulcers (DU); anti-RNA polymerase III antibodies have been reported in patients with dcSSc and scleroderma renal crisis. Other common disease features include constitutional symptoms, such as fatigue and weight loss, musculoskeletal inflammation and gastrointestinal involvement (2).

Despite a broad knowledge of SSc clinical manifestations, the initial pathogenic processes are still poorly understood. This illness is characterized by different interactions between cells, cytokines and extracellular matrix (ECM), that lead to immune system activation, vascular damage and excessive synthesis and deposition of normal collagen from activated fibroblasts, the final effectors in this disease (4).

### TGFβ involvement in SSc

Among the potential pathogenic cytokines that have been studied over time, the Transforming Growth Factor β (TGFβ) superfamily is considered pivotal because of its stimulatory properties on fibroblasts and matrix production (5). In addition, TGFβ is perhaps the most potent inducer of Connective Tissue Growth Factor/Cellular Communication Network factor 2 (CTGF/CCN2), a hallmark of fibrotic pathologies including SSc, thus far identified (6).

The TGFβ superfamily of ligands includes the families: TGFβ, Bone Morphogenetic Proteins (BMPs), Growth Differentiation Factors (GDFs), activins and inhibins. Receptors involved in the pathway are classified in type I, II and III. Type I receptors include Activin Like Kinase 1 to 7 (ALK1-7), type II receptors include TbRII, ActRII, ActRIIB, AMHRII and BMPRII, type III receptors include Betaglycan and Endoglin (ENG) (7).

Signal transduction foresees as a first step the binding of the ligand (in an homodimeric complex) to a complex formed by two type II receptors, which in turn recruit two type I receptors. The whole complex is stabilized by an homodimeric type III receptor complex. The phosphorylation cascade starting from receptors activates SMAD proteins complexes (SMAD2/3 or SMAD1/5), which recruit and activate SMAD4, translocate to the nucleus and, together with other transcription factors, regulate target genes expression (8).

**Figure 1:**
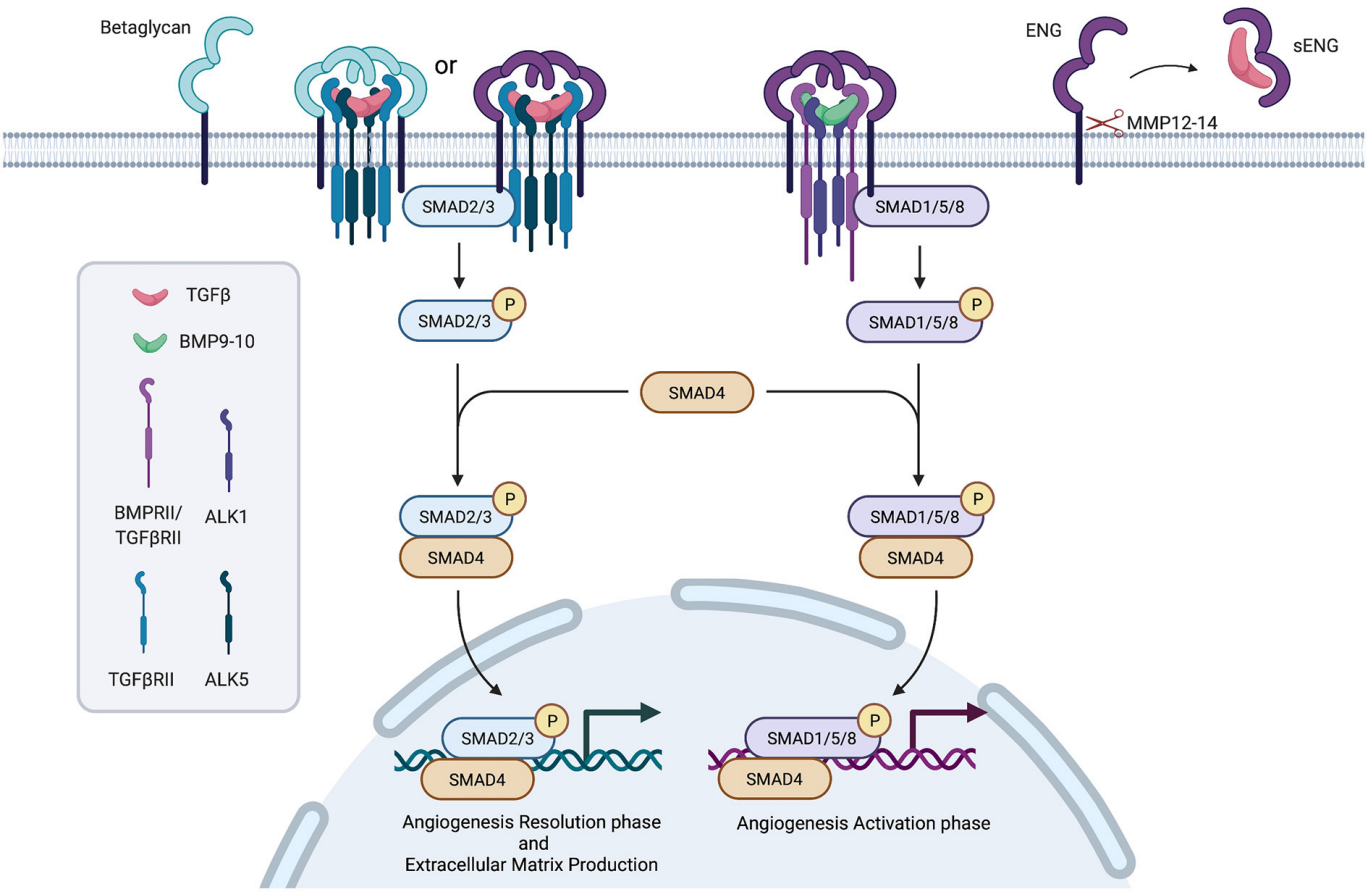
Simplified representation of the TGFβ/ENG pathway. Created with BioRender.com.

Differences in type I and II receptors expression further modulate signal transduction (Figure 1).

It has been supposed a role for TGFβ receptors in SSc pathogenesis and a crucial player in modulating TGFβ signal transduction has been suggested for the type III receptor, Endoglin.

### The TGFβ coreceptor Endoglin

Endoglin, also known as Cluster of Differentiation 105 (CD105), is encoded by *ENG*, located on chromosome 9q34.11 (9).

It is mainly expressed in endothelial cells (ECs) and activated monocytes but also in mesenchymal cells and in some cancerous cells as well as in some immune cells subpopulations (10).

Alternative splicing acting on *ENG* primary transcript is responsible for the production of two different isoforms: long (L)-endoglin and short (S)-endoglin.

L-ENG is considered the canonical isoform (NM_001114753.3; ENST00000373203.9); it is composed of 15 exons and it produces a protein that counts 658 amino acids.

S-ENG is the minor isoform (ENST00000344849.4) which differs from L-ENG because intron 14 is retained, in which a stop codon leads to the formation of a shorter protein of 625 residues.

Both these isoforms encode for a transmembrane glycoprotein that participates to the TGFβ signaling pathway as a type III receptor homodimeric complex (11).

The two monomers interact through multiple disulfide bridges between cysteines to form the homodimeric structure.

Each 95kDa monomer is composed of: (1) a 561-amino acid extracellular domain that contains a N-terminal orphan domain (composed of OR1 and OR2 regions), linked to a proximal Zona Pellucida domain containing the RGD tripeptide fundamental for cellular adhesion; (2) a 24-residues long transmembrane domain; (3) a cytoplasmic domain with a length of 47 amino acids in L-ENG that terminates with a PDZ-binding motif and a 14 residues length in S-ENG which lacks the PDZ-binding motif (12–15).

In particular, L-ENG recruits BMPs or TGFβ to facilitate the formation of the heterotetrameric complex composed of ALK1 receptor and type II receptor, signaling through SMAD1/5. This stimulates angiogenesis and cell proliferation. On the other hand, S-ENG recruits TGFβs mediating the formation of the ALK5/TbRII complex, activating SMAD2/3 and leading to the inhibition of angiogenesis and cell proliferation, and senescence induction (15, 16).

The membrane-proximal portion of the extracellular domain may undergo proteolytic shedding mediated by Matrix MetalloProteinase-14 and−12 (MMP-14 and MMP-12), which are responsible for the release of a soluble Endoglin form (sENG) (17, 18).

Endoglin is a key player in angiogenesis and vascular maintenance. Pathogenic variants leading to haploinsufficiency in this gene are responsible for Hereditary Hemorrhagic Telangiectasia type 1 (HHT1), a rare autosomal dominant vascular disorder, among whose features are the mucocutaneous telangiectases (19). Braverman et al. in 1990 underlined that HHT and SSc telangiectases are “clinically indistinguishable” (20).

On the other hand, high circulating sENG levels have been observed in preeclampsia, in endothelium-associated tumors, in sera of patients with liver fibrosis and in the interstitium in human renal fibrosis (21–26).

The simultaneous presence of an altered ENG expression pattern and phenotypic manifestations resembling SSc suggests a role of this protein in the disease-related mechanisms. We conducted a systematic review with the objective to collect bibliographic data regarding the topic, merging pieces of information that had never been considered together before. We aimed to process data in order to classify them into clinical and cellular fields, with the final purpose of re-combining and re-elaborate the acquired findings. This way we could highlight a correlation between a dysregulated ENG expression and both the clinical manifestations and cellular alterations observed in SSc, also suggesting novel hints for future studies.

## Methods

### Identification of studies and search strategy

This systematic review was conducted according to the Preferred Reporting Items for Systematic Reviews and Meta-Analyses (PRISMA) guidelines (27).

To assess inclusion parameters, we exploited two operators (OR and AND), that were used in order to include records that contained at least one keyword concerning endoglin (“Endoglin,” “CD105,” “sENG”) and at least one keyword concerning the disease (“Systemic Sclerosis,” “Scleroderma,” “SSc,” “CREST”) in all paper fields.

We searched for studies on the electronic databases MEDLINE (*via* PubMed), Embase and Web of Science. We performed a single literature search on November 2nd, 2021.

Search strings were designed according to the indications given by each database.

Pubmed: [(endoglin) OR (CD105) OR (sENG)] AND [(systemic sclerosis) OR (scleroderma) OR (SSc) OR (CREST)] in All Fields.

Embase: (“endoglin” OR “CD105” OR “sENG”) AND (“systemic sclerosis” OR “scleroderma” OR “SSc” OR “CREST”).

Web of Science: {ALL=[(endoglin) OR (CD105) OR (sENG)]} AND {ALL=[(systemic sclerosis) OR (scleroderma) OR (SSc) OR (CREST)]}.

### Eligibility criteria and study selection

We decided to include both articles and abstracts, without a related publication.

The following inclusion criteria were assessed: (1) articles focused on the relationship between endoglin and scleroderma; (2) articles in English language.

The following exclusion criteria were assessed: (1) articles dealing with endoglin exclusively as a cellular biomarker; (2) articles that were not focused on endoglin-SSc relationship, although both subjects were included; (3) review articles that exclusively cited other publications—already included in our revision—without adding novel data; (4) abstracts published by different authors from the same group and/or in different congresses but reporting the same data—only one of the similar abstracts has been selected for being cited in this review (the less recent).

The author GS conducted the selection in duplicate using Endnote X9 software. Duplicates were removed first automatically and then manually.

In a second step AS, CN and CO screened articles to verify the actual presence of keywords and to eliminate records that included some of the keywords in non-pertinent contexts (ex. “iliac crest,” “neural crest,” “Seng Hospital,” “SSc = Skeletal Stem Cell,” “Multiple Sclerosis”).

References cited in key publications selected from databases were also manually searched by authors to identify any additional published literature.

The subsequent step involved the selection of the remaining reports using the aforementioned inclusion/exclusion criteria.

Eligibility was assessed independently by each of the eight authors. As this systematic review holds papers with both clinical and/or molecular topics, the authors divided them as follows: clinical arguments were evaluated by SG, BLP and LC and molecular ones by AS, CC, CN and CO, and many records were evaluated by both working groups. Any disagreements between the review team members were resolved through discussion until consensus was reached. Results were summarized by author, using a spreadsheet.

## Results

**Figure 2:**
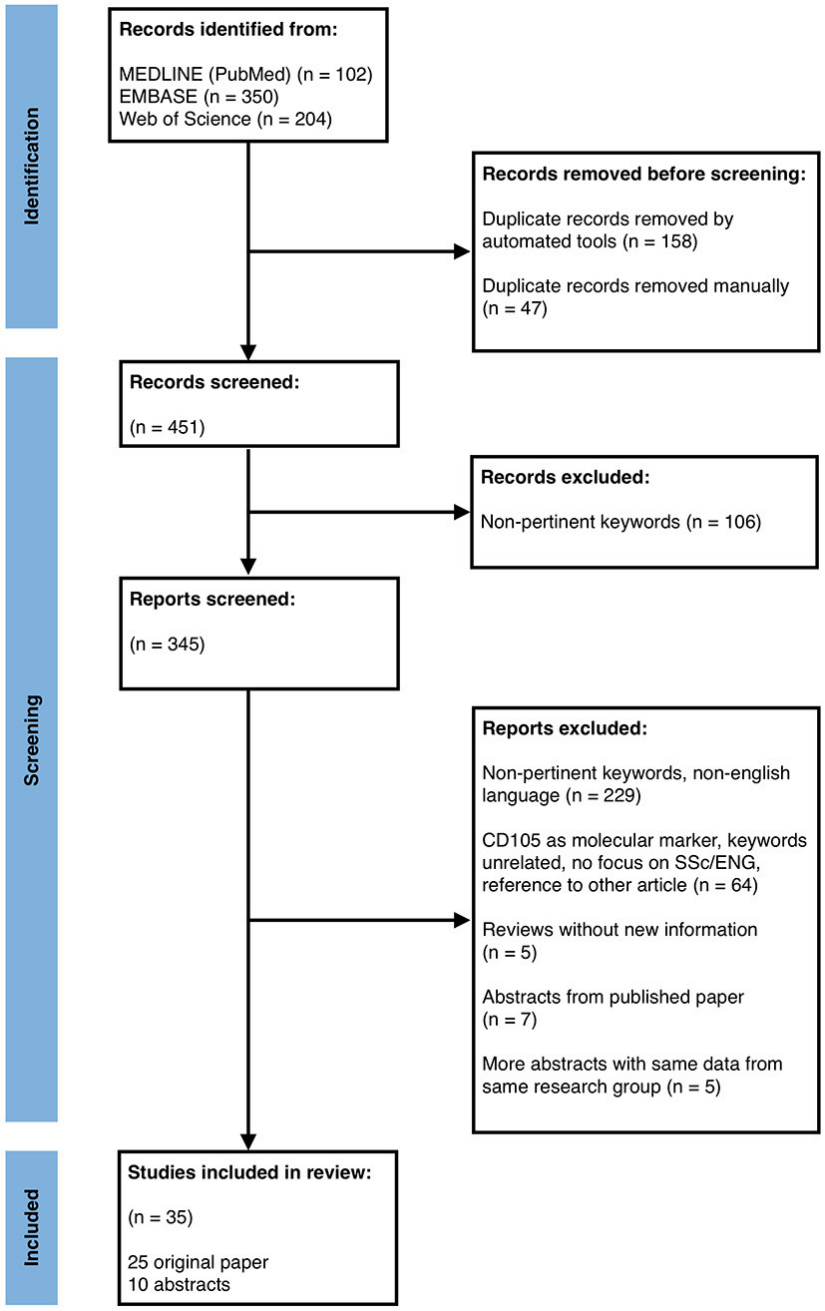
PRISMA flow diagram depicting the different phases of records identification, inclusion and exclusion for the systematic review.

The search provided a total of 656 results. In the PRISMA flowchart reported in Figure 2 are described the identification, screening and inclusion steps.

At the end, 25 original papers and 10 conference abstracts passed the selection and were studied and summarized by author (Table 1). We are aware of possible missing results, although we tried to minimize this bias as all the authors have double checked each article/abstract independently.

**Table 1:** List of studies selected for the systematic review.

**References**	**Title**	**Type**	**Year**	**Topic**	**ENG Type**
Alzahrani et al. (28)	Endoglin haploinsufficiency is associated with differential regulation of extracellular matrix production during skin fibrosis and cartilage repair in mice	Article	2018	M	ENG
Avouac et al. (29)	Correlations between angiogenic factors and capillaroscopic patterns in systemic sclerosis.	Article	2013	C	sENG
Cambon et al. (30)	Immune properties and anti-fibrosis effect of mesenchymal stroma cells in systemic sclerosis.	Abstract	2013	M	ENG
Cambon et al. (30)	Mesenchymal stromal cells in refractory systemic sclerosis: Anti-fibrosis property as adjuvant therapy?	Abstract	2013	M	ENG
Ciurzyński et al. (31)	Serum endothelin-1 and NT-proBNP, but not ADMA, endoglin and TIMP-1 levels, reflect impaired right ventricular function in patients with systemic sclerosis	Article	2013	C	sENG
Coral-Alvarado et al. (32)	Potential biomarkers for detecting pulmonary arterial hypertension in patients with systemic sclerosis	Article	2009	C	sENG
Coral-Alvarado et al. (33)	Serum endoglin levels in patients suffering from systemic sclerosis and elevated systolic pulmonary arterial pressure.	Article	2010	C	sENG
Del Papa et al. (34)	Bone marrow endothelial progenitors are defective in systemic sclerosis	Article	2006	M	ENG
Del Papa et al. (35)	Mesenchymal stem cells from bone marrow of patients with systemic sclerosis: Properties and pathogenic perspectives	Abstract	2009	M	ENG
Dharmapatni et al. (36)	The TGF beta receptor endoglin in systemic sclerosis	Article	2001	C/M	sENG/ENG
Fujimoto et al. (37)	A clue for telangiectasis in systemic sclerosis: Elevated serum soluble endoglin levels in patients with the limited cutaneous form of the disease	Article	2006	C	sENG
Gerlicz-Kowalczuk et al. (38)	Serum endoglin level in patient with systemic sclerosis fibrosis	Abstract	2020	C	sENG
Holmes et al. (6)	Elevated CCN2 expression in scleroderma: a putative role for the TGF beta accessory receptors TGF beta RIII and endoglin	Article	2011	M	ENG
Hummers et al. (39)	Circulating inhibitors of angiogenesis in scleroderma	Abstract	2009	C	sENG
Jouvray et al. (40)	Whole-Body Distribution and Clinical Association of Telangiectases in Systemic Sclerosis	Article	2018	C	sENG
Kolstad et al. (41)	Detection score for pulmonary hypertension in systemic sclerosis patients: Observations from the pharos registry	Abstract	2020	C	sENG
Kudo et al. (42)	Decreased Interleukin-20 Expression in Scleroderma Skin Contributes to Cutaneous Fibrosis	Article	2014	M	ENG
Leask et al. (43)	Dysregulation of transforming growth factor beta signaling in scleroderma - Overexpression of endoglin in cutaneous scleroderma fibroblasts	Article	2002	M	ENG
Márquez Fernández et al. (44)	Relationship between biological biomarkers and changes in right ventricle that precede pulmonary hypertension in patients with systemic sclerosis	Abstract	2017	C	sENG
Mecoli et al. (45)	The Utility of Plasma Vascular Biomarkers in Systemic Sclerosis: A Prospective Longitudinal Analysis	Article	2020	C	sENG
Mecoli et al. (46)	Vascular biomarkers and digital ulcerations in systemic sclerosis: results from a randomized controlled trial of oral treprostinil (DISTOL-1)	Article	2020	C	sENG
Mertens et al. (47)	The identification of CCL18 as biomarker of disease activity in localized scleroderma	Article	2019	C	sENG
Morris et al. (48)	Endoglin Promotes TGF-beta/Smad1 Signaling in Scleroderma Fibroblasts	Article	2011	M	ENG
Rodriguez Fraga et al. (49)	Potential role of biomarkers and cardiac imaging in scleroderma patients with subclinical myocardiopathy	Abstract	2015	C	sENG
Schiopu et al. (50)	Prevalence of subclinical atherosclerosis is increased in systemic sclerosis and is associated with serum proteins: a cross-sectional, controlled study of carotid ultrasound	Article	2014	C	sENG
Silva et al. (51)	Impaired angiogenesis as a feature of digital ulcers in systemic sclerosis	Article	2016	C	sENG
Silva et al. (52)	Endothelial Dysfunction and Nailfold Videocapillaroscopy Pattern as Predictors of Digital Ulcers in Systemic Sclerosis: a Cohort Study and Review of the Literature	Article	2015	C	sENG
Silva et al. (53)	Predictive value of vascular disease biomarkers for digital ulcers in systemic sclerosis patients	Article	2015	C	sENG
Silva et al. (54)	Microvascular damage, endothelium dysfunction and angiogenesis biomarkers as predictors of digital ulcers in systemic sclerosis	Abstract	2014	C	sENG
Silva et al. (55)	Peripheral vasculopathy in Raynaud phenomenon: Vascular disease biomarkers	Article	2016	C	sENG
Trinder et al. (56)	A Putative Role for the TGF beta Accessory Receptors Betaglycan and Endoglin in pulmonary Complications of Scleroderma	Abstract	2012	M	ENG
Walker et al. (57)	Histopathological and ultrastructural features of dermal telangiectasias in systemic sclerosis	Article	2005	C	ENG
Wienke et al. (58)	Biomarker profiles of endothelial activation and dysfunction in rare systemic autoimmune diseases: Implications for cardiovascular risk	Article	2021	C	sENG
Wipff et al. (59)	Disturbed angiogenesis in systemic sclerosis: high levels of soluble endoglin	Article	2008	C	sENG
Wipff et al. (60)	Association between an endoglin gene polymorphism and systemic sclerosis-related pulmonary arterial hypertension	Article	2007	C/M	ENG

*ENG, Endoglin; sENG, soluble Endoglin; C, clinical; M, molecular and cellular; C/M, clinical/molecular and cellular*.

### Cellular and molecular findings

Our search found 8 articles and 4 conference abstracts (Table 1, label “M” in column “Topic”) about the relation of Endoglin and SSc in a cellular and molecular context and, as expected, different cellular subsets provided different results.

#### *In vivo* studies

We found a single cross sectional study, in which immunohistochemistry on skin biopsy sections of 10 SSc patients (6 dcSSc and 4 lcSSc) and 6 controls (3 “inflammatory”- subacute eczema, lichen planus and papular urticaria- and 3 normal) showed a significant upregulation of Endoglin expression on endothelial cells of SSc and inflammatory subjects compared to HCs (36). They also analyzed by flow cytometry purified monocytes from peripheral blood of 10 additional SSc patients (3 dcSSc, 5 lcSSc, 2 overlap) and 17 HCs, and did not find any significant difference in Endoglin expression (36).

#### *In vitro*/*ex vivo* studies

##### Stem precursor cells

A focus on stem/precursor cells was published in 2006 by Del Papa and colleagues. When they investigated circulating endothelial progenitor cells phenotype in bone marrow (BM) using flow cytometry, they observed a significant Endoglin overexpression in SSc patients vs. controls. Subsequently, authors analyzed bone marrow stromal cells without finding any difference in Endoglin expression between patients and controls. Instead, immunocytochemical analysis of *in vitro* differentiated ECs from precursors, once analyzed for the presence of endothelial markers, showed, among others, a higher expression of Endoglin even before activation by IL-1α (34).

The same group demonstrated an increased Endoglin surface expression in Nerve Growth Factor Receptor- positive (NGFR^+^) SSc BM-derived Mesenchymal Stem Cells (BM-MSCs) (35).

The group by Cambon and collaborators further investigated MSCs subtypes describing their phenotypic and functional characteristics. The authors demonstrated that, after stimulation by Vascular Endothelial Growth Factor (VEGF) or Endothelin-1 (ET-1, an important mediator of vascular disease in SSc, inducer of collagen in fibroblasts), both BM-MSC and MSC from SSc patients expressed more ENG than controls (30).

##### Fibroblasts

The vast majority of data investigating Endoglin role in SSc relies on fibroblasts derived from patients' skin biopsies (6, 43, 48).

Leask et al. (43) compared fibroblasts from skin samples of 8 dcSSc subjects to 6 HCs and described for the first time a significantly higher expression in SSc at the mRNA level of both collagen type IV and ENG. Western Blot analysis confirmed ENG overexpression in SSc patients at the protein level.

The authors also noted differences in SSc fibroblasts phenotype. In fact, immunohistochemistry results showed that normal fibroblasts lacked Endoglin staining (with ENG expression confined to a limited number of cells), while SSc fibroblasts showed a bright membrane-localized signal consistent with a generalized overexpression of Endoglin throughout the cell population. Fluorescence Activated Cell Sorting (FACS) confirmed these results and evidenced an augmented number of TGFβ receptors on the SSc fibroblasts surface (43). Many years later, the same group (6) published additional findings on the previously analyzed fibroblasts primary cell lines, reporting a significant higher cell surface expression of Betaglycan in SSc.

ENG overexpression in SSc fibroblast was later confirmed by Morris et al. together with collagen type I, CTGF/CCN2, pSMAD1. Of note, ALK1 levels were not altered (48).

Additional experiments further investigated effects of TGFβ induction on *ENG* hyperexpression/inhibition in fibroblast cultures from patients, controls and in the NIH3T3 mouse lineage.

Morris et al. (48) assessed the effects of exogenous TGFβ on the expression of Endoglin in normal fibroblasts without finding any significant activation either at transcriptional or translational level.

Leask et al. (43) observed that the *ENG* overexpression, induced by a transfection vector in NIH3T3 and primary human dermal fibroblasts suppressed the ability of TGFβ1 to induce CTGF/CCN2 promoter activity. However, it was not able to suppress the ability of SMAD3 and SMAD4 to activate the CTGF/CCN2 promoter.

Into NIH3T3 fibroblasts they also observed the inhibition of the TGFβ-stimulated accumulation of activated nuclear SMAD3 (43).

In 2011, Holmes et al., following their observation of a higher expression of Betaglycan in SSc fibroblast, overexpressed Betaglycan gene or *ENG* or both in NIH3T3 cells and tested their capacity to modulate the TGFβ induction of the CTGF/CCN2 promoter. They assessed that in the first case Betaglycan enhanced both basal and TGFβ1 induced activity, ENG suppressed the induction of TGFβ1, while together they led to a significant increase in basal and TGFβ1 induced activity (6).

In 2012, the same group presented similar results on SSc-lung fibroblasts, which showed overexpression of Betaglycan and Endoglin; when a transient transfection further increased the levels of these accessory receptors, they inferred an altered cellular response to TGFβ, by quantifying the expression of fibrogenic genes (56).

Evidence on the role of Endoglin in regulating ECM production was reported by Morris et al. (48), who inhibited *ENG* using a short hairpin RNA adenovirus (siENG) and searched for altered expression of COL1A1, COL1A2, CTGF/CCN2, pSMAD1 and pSMAD3. They observed that in the majority of SSc fibroblasts basal expression of Endoglin, pSMAD1, pSMAD3, CCN2, collagen type I was already elevated and was reduced after treatment with siENG, while in normal fibroblasts this treatment did not lead to significant changes other than in Endoglin, as expected.

Similar experiments inhibiting ALK1 suggested that Endoglin/ALK1 signaling is responsible for the constitutive activation of SMAD1 signaling in SSc fibroblasts. Moreover, co-immunoprecipitation studies identified an increased level of Endoglin/ALK1 complexes in SSc fibroblasts strains. Authors also demonstrated that the Endoglin/ALK1 pathway can regulate the ET-1 gene expression in dermal fibroblasts, supporting the pro-fibrotic contribution of Endoglin/ALK1 axis in SSc (48).

##### IL-20

The levels of IL-20, which is expressed by multiple cell types (i.e., monocytes, skin keratinocytes) and implicated in autoimmune diseases pathogenesis (61), were found to be reduced in the involved skin of SSc patients by Kudo and collaborators in 2014. They also reported that the addition of exogenous IL-20 decreased ENG and SMAD3 mRNA levels in normal and SSc fibroblasts after induction with TGFβ. Similar results were obtained when IL-20-stimulated normal fibroblasts were compared to untreated cells by PCR array: IL-20 induced expression and protein synthesis of the FLI1 transcription factor, while SMAD3 and ENG are significantly downregulated (42).

##### Animal models

Seeking for animal models, the only available *in vivo* data come from the paper of Alzahrani et al. (28), who studied the SSc mouse model (bleomycin-induced skin fibrosis) in the genetic background of Endoglin haploinsufficiency. They observed that Eng^+/−^ mice were more resilient to bleomycin-induced skin fibrosis than Eng^+/+^ mice and displayed diminished basal and bleomycin-induced ECM protein expression. Endoglin haploinsufficiency did not alter proliferation rate or SMAD phosphorylation but enhanced plating efficiency of primary mouse dermal fibroblasts *in vitro*. Moreover, they assessed that chondrocytes isolated from Endoglin heterozygous mice showed decreased Endoglin (as expected) and increased type II collagen levels (28).

### Clinical findings

Our search found 19 articles and 6 conference abstracts (Table 1, label “C” in column “Topic”) regarding clinical manifestations and relations between serum Endoglin concentrations (sENG, although it is not always specified) and SSc.

#### General disease characteristics

Four studies found higher sENG in SSc patients compared to HCs (38, 39, 59), only one of them initially described sENG concentrations similar in SSc, systemic lupus erythematosus (SLE) and HCs (37) but, after stratification for HCs' normal sENG concentration, patients with lcSSc showed higher values of sENG compared to dcSSc, SLE and HCs. This last difference was not confirmed in a study on a small group of 26 SSc vs. 10 HCs (38). Two studies did not find differences in sENG concentrations between SSc and HCs, although both of them did not stratificate SSc patients for disease characteristics (29, 36).

The first group of authors that in 2006 tried to relate sENG concentrations to SSc clinical manifestations described that higher values of sENG were related to telangiectases, ACA positivity, anti-topoisomerase I antibodies negativity and higher pulmonary artery systolic pressure (PAPs) scores, while there was no correlation with cardiac, esophageal or renal involvement (37).

These findings were partially confirmed 2 years later by a study on 187 SSc patients, in which sENG levels were found to be higher in ACA positive SSc, in patients with digital ulcers and in patients with lung fibrosis, expressed as carbon monoxide diffusing lung capacity divided by the alveolar volume resulting <75% (DLCO/VA <75%). Also multiple linear regression highlighted a significant relation between skin involvement and higher sENG values for DU, ACA positivity and reduced DLCO/VA (59). These results were similar to the ones in Hummers', which found that sENG concentrations were inversely related to DLCO (39), but dissimilar from a study on a little group of 26 SSc that reported an association between higher levels of sENG and gastrointestinal involvement, while lower levels were associated to the progression of lung fibrosis (38).

Hummers also described a correlation between ACA positive SSc, Raynaud's Phenomenon (RP) and higher sENG concentrations (39).

Wipff et al. showed that sENG concentrations were comparable between SSc patients with or without PAH and also between SSc patients with “HHT-like” vascular phenotype (watermelon stomach or telangiectases) and other SSc patients or controls. The same authors, in a different paper, studied the 6bp insertion polymorphism of the *ENG* gene (*ENG* c. 991+21_991+26dupCCTCCC; rs148063362) in both SSc patients and HCs and found only a significantly lower frequency of this genotype in 29 SSc-PAH patients compared both to 140 HCs and 251 SSc without PAH (59, 60). Of note, this is the only group of researchers that aimed at a “genetic investigation” in SSc and ENG.

Several studies addressed the relationship between sENG concentrations and cutaneous microvasculopathy in SSc. Interestingly, in two studies that evaluated concentrations of cytokines and chemokines related to endothelial dysfunction, angiogenic homeostasis and tissue inflammation in patients with connective tissue diseases (CTD) comprehensive of LoS, sENG was never elevated, both in active and inactive disease (47, 58).

#### Microvascular manifestations

The differences in endothelial dysfunction biomarkers and vascular disease parameters were also assessed by Silva et al. in a cohort of 32 patients with primary RP and 77 SSc patients with secondary RP, of which 38 patients had severe DUs. Endoglin serum levels were found to be higher in SSc patients with secondary RP and DU compared to primary RP and to secondary RP without DU (55).

Different authors investigated a putative role of sENG in predicting the development of DU. Mecoli et al. carried out a prospective cohort study of 300 patients with SSc, lacking evidence of pulmonary hypertension (defined as an estimated right ventricular pressure of <40 mmHg by transthoracic echocardiography—PH) and/or active DU at enrollment, that underwent clinical assessments and plasma samples collection at 6–12 month intervals for at least a 5-year period. Forty-six (15%) patients developed PH and sixty-nine (23%) patients developed DU. There was no significant difference in vascular biomarkers based on patient comorbidities, medications, disease duration, age at enrollment or age at diagnosis. sENG levels measured at cohort entry were not associated with the development of PH or DU; however, sENG measurement at time point 2 (obtained 2.8 ± 2.5 years prior to DU) was significantly related to the occurrence of DU (45). These data were not confirmed in another work led by Mecoli and colleagues in the setting of a multicenter randomized controlled trial evaluating the efficacy of treprostinil diolamine (DISTOL-1). The authors examined serum levels of sENG and other 18 vascular, angiogenic and inflammatory biomarkers in 124 SSc patients to determine if any specific biomarker could predict meaningful outcomes associated with DU. Over the 20-week trial, 66% of patients had their largest or most painful DU completely healed, 44% developed new ulcers, 58% had complete healing of all DU. However, after adjusting for multiple comparisons, no individual biomarker including sENG was significantly related to any clinical outcome of interest (46). A prospective analysis on various SSc cohorts highlighted that increased sENG concentrations were related to active DU and to NVC (Nailfold Videocapillaroscopy) alterations with scleroderma pattern late (54). Nevertheless, sENG was not found to be a predictive factor for the development of a first episode of DU or even for the recurrence of new DU in the follow-up (51–53).

Concerning telangiectases, a large study involving 106 SSc patients sought to describe the whole-body distribution of telangiectases and the associations with clinical and biological manifestations and found that almost every patient (92.5%) had at least 1 telangiectasia, and half of them (51.9%) had at least 1 telangiectasia larger than 5 mm. The median telangiectases number was 30 and telangiectases were mostly distributed on the face (37.2%), hands (26.4%), and upper part of the trunk (17.1%). Regarding factors associated to telangiectases, the authors found that sENG concentrations, male sex, pulmonary hypertension, history of pulmonary embolism and decreased glomerula filtration rate (GFR) were independently associated with the total number of telangiectases. The number of hands or face telangiectases, moreover, was well-correlated with the total telangiectases number and could be useful to identify patients with SSc who require closer monitoring for PH (40). Walker et al. analyzed the immunohistological and ultrastructural features of established telangiectases in long-standing limited scleroderma, and found benign features, such as thickened collagen fibers in the reticular and deep dermis layers with scarce infiltration of inflammatory cells. No enhanced endothelial staining was obtained with antibodies directed against Endoglin, suggesting a resting state of the lining endothelium (57).

#### Cardiovascular manifestations

As PAH and cardiac disease are one of the consequences of SSc that mainly affects patient's survival, quite a number of authors tried to relate sENG concentrations to cardiovascular impairment.

Schiopu et al. focussed their research on the evaluation of subclinical atherosclerosis and carotid intimal media thickness (IMT) in SSc patients compared with HCs, and highlighted that sENG concentrations, together to other serological markers of vasculopathy and fibrosis, were related to the presence of atheromatous plaques and subclinical atherosclerosis while not association was found with carotid IMT (50).

Regarding right heart involvement only serum concentration of the pro-brain natriuretic peptide N-terminal fragment (nT-proBNP) and ET-1 have been found to be higher in SSc patients compared to HCs, while no difference was found with asymmetric dimethylarginine (ADMA), sENG and human tissue inhibitor of Matrix Metalloproteinase (TIMP-1) concentrations. Moreover, none among ADMA, sENG and TIMP-1 has been found to be related with ultrasound findings of right heart overload (31).

When evaluating cardiac fibrosis with magnetic resonance (MRI) and cardiac ultrasonography some authors found that sENG concentrations were related to high sensitive cardiac T-troponin and systolic eccentricity index >1, while no correlation was found between serological biomarkers and right ventricle thickness (44, 49).

A cross-sectional study tried to determine if serum levels of different biomarkers in 20 patients with SSc and PAH in comparison with 20 HCs could have a potential role as predictors of PAH and found that mean sENG levels were significantly higher in the SSc-PAH group, even if there was not a correlation with the mean systolic pulmonary artery pressure (PAPs) (32). The same authors subsequently measured sENG concentrations in SSc-PAH patients compared with SSc without PAH (20 + 20 patients) and 20 HCs and found a slight elevation without statistical significance in SSc-PAH compared to SSc without PAH, while the difference was significant with HCs. Also after this stratification, no correlation was demonstrated between sENG and PAPs (33).

The authors, moreover, found a correlation between serum concentrations of ET-1 and ENG and between IL-8 and ENG and proposed a possible role of these biomarkers as diagnostic tools for PAH (32).

A recent study from the PHAROS Registry tried to understand if endothelial biomarkers as ENG and Pentraxin 3 (PTX-3) could detect or be predictive for PAH: of 558 SSc patients the Registry had 118 blood samples and among them they did not find significant differences between SSc-PAH and SSc without PAH (41). No correlation between sENG levels and development of PAH was also recorded in a cohort of 300 SSc patients from Mecoli and colleagues (45).

## Discussion

This systematic review was designed to highlight the clinical and molecular role of Endoglin in a rare connective tissue disease whose pathogenesis is only partially known, Systemic Sclerosis.

Concerning the possible limits of our search, we cannot exclude that the use of boolean operators in our strings was uneffective in finding all the available papers in the field. However, the use of different strings in different databases should have reduced this issue at a minimum. Another bias can be represented by the selection step, in which we excluded many articles based on the role of Endoglin as a histological vascular marker rather than a signaling receptor. Also in this case, we tried to reduce this risk by performing the selection independently by each author and discussing disagreements.

The first observation regarding the analyzed clinical studies is that most of them refer to data obtained on sENG, while the molecular ones preferentially investigated Endoglin as a membrane-bound protein.

The reviewed articles suggest that some clinical characteristics of SSc are related to higher sENG concentrations such as ACA positivity, limited cutaneous disease, telangiectases, digital ulcers and lung fibrosis, but these results are not conclusive since some authors found completely different data (29, 41, 45).

A role of Endoglin in systemic fibrosis can be suggested since sENG concentrations have never been reported high in LoS (47, 58), which is characterized by skin-limited fibrosis without internal organ involvement. A similar inference can be made regarding its role as a marker of vasculopathy, as sENG concentrations have been reported higher in secondary RP compared to primary RP (55), considering that the primary form is benign, spontaneously resolving and rarely associated with development of a systemic disease. Furthermore, sENG was also found high in patients with atheromatous plaques and subclinical atherosclerosis, which are vasculopathy manifestations and important cardiovascular risk factors (50).

Moreover, SSc manifestations that seem to be related to higher sENG concentrations could be all grouped in the former CREST syndrome, which encompasses HHT as differential diagnosis.

HHT is a rare genetic disease leading to vascular malformations, caused by pathogenic variants in *ENG* or *ACVRL1*, the two major genes. In this field, Wipff and collaborators described no sENG differences between “HHT-like” SSc and other SSc or HCs, nor found differences in frequency of the 6bp insertion polymorphism of the *ENG* gene into these populations (59, 60).

An upregulation of ENG is the main result of most of the articles reporting cellular and molecular data in SSc patients compared to HCs. Notably, none of the published data takes into account the two different ENG isoforms, L-ENG and S-ENG, which are known to have distinct biological roles (62).

Endoglin is known to be a marker of activated endothelial cells (12) and one of the first evidences of this role has been produced after the analysis of SSc-involved skin endothelium, where it was found to be overexpressed (36).

The higher amount of ENG observed also in SSc fibroblasts seems to be more pronounced at the protein, rather than mRNA, level and the suggestion is that post-transcriptional mechanisms could enhance protein expression in SSc fibroblasts (43). Our search did not provide any result regarding either this process, or an altered expression of miRNAs in SSc regulating *ENG* (63), making this an interesting field of future investigation.

As a consequence of Endoglin overexpression in SSc fibroblasts, numerous abnormalities of the TGFβ signaling pathway were observed (64).

*In vitro* data obtained from forced ENG expression in fibroblasts showed that ENG may suppress TGFβ1 signaling pathways upstream SMADs (43) and similar experimental data lead to the hypothesis that the ALK1 and ALK5 balance, in which ENG is the pivotal TGFβ co-receptor, is altered (48).

Previous studies have supported, with contrasting results, a positive role of Endoglin in fibrosis (65, 66). In this context, Morris et al. showed that ENG inhibition has no significant effect on fibrotic protein production in normal fibroblasts. However, constitutively high levels of ENG were found in complex with ALK1 in SSc fibroblasts and were associated with a fibrotic phenotype. Endoglin and pSMAD1 are therefore required for ECM production in SSc fibroblasts. Authors also compared ALK1 and ENG inhibition and observed a less pronounced suppressive effect on ECM genes by the first, suggesting a pro-fibrotic function for ENG, which could be independent from its role in the ALK1-induced SMAD1 phosphorylation (48). This hypothesis is further supported by a modest increase of collagen and CTGF/CCN2 production in both SSc and normal fibroblasts when the ALK1/SMAD1 signaling is activated (48).

On the other hand, we can highlight the contribution of the ALK1/ENG axis to an abnormal ET-1 production in SSc when ENG or ALK1 are inhibited and there is a reduced ET-1 stimulation by TGFβ.

Taken together, the results of Morris et al. suggest a positive regulation by Endoglin in ECM production in SSc fibroblasts and the final hypothesis is that activation of the TGFβ/ALK1/ENG pathway may have an indirect pro-fibrotic effect in SSc *via* induction of ET-1 (48).

In addition, a significant role played by ENG in regulating CTGF/CCN2 activity in a ligand-independent manner has been suggested by Holmes et al. (6).

Interestingly, some authors tried to correlate Endoglin expression in fibroblasts with disease progression and suggested that it may represent a relatively late response in the development of fibrosis in SSc lesions (43).

Considering the reported findings, we have to mention that *in vitro* ENG expression was found altered by changing experimental settings (i.e., the purification methods and temperature) (36).

Taken together, these results propose a scenario in which SSc fibroblasts present different basal characteristics when compared to normal fibroblasts and, among them, SSc fibroblasts express higher Betaglycan levels (6), thus causing an excessive response to TGFβ stimulation and a consequent overproduction of pro-fibrotic proteins (6, 48). As a response, cells produce more ENG which can hijack TGFβ from the Betaglycan pathway. This is achieved both by the receptor form—and therefore promoting the ENG/ALK1/SMAD1, in spite of ALK5/SMAD2/3 pathway—both by the soluble form—capturing the circulating ligand (59, 62). In this way, ENG overexpression can suppress, although not completely, TGFβ induction of ECM genes, as seen in both human fibroblasts and NIH3T3 cells. However, a TGFβ/ALK1/ENG pathway activation may have an indirect pro-fibrotic effect in SSc *via* induction of ET-1 (48).

All these hypotheses, however, do not take into account the opposite role of the two alternative splicing ENG-isoforms: L- and S-ENG, and none of the studies used experimental procedures to differentiate the isoforms. This lack of knowledge reduces our understanding of the Endoglin role in SSc physiopathology. Indeed, it is known that L-ENG promotes TGFβ1-induced cellular proliferation, whereas S-ENG increases Collagen I and CTGF/CCN2 expression and reduces cell proliferation (16, 67). Moreover, the two ENG isoforms mutually activate either the ALK1/SMAD1 (L-ENG) or the ALK5/SMAD2/3 (S-ENG) pathway. The aforementioned hypothesis of an ENG overexpression as a compensatory response by SSc cells can be further enriched by studies about S-/L-ENG ratio in patients along disease progression. Of note, it has been clearly demonstrated that, physiologically, this ratio increases with age (68).

It is also important to mention that another player in the S-/L-ENG ratio is the alternative splicing factor or splicing factor-2 (ASF/SF2), which favors the synthesis of S-ENG (62). There is no data on expression of this protein in SSc and this can be another field of investigation for a better understanding of disease pathogenesis.

## Conclusions

Our systematic review is, to date, the first one that specifically aims to discuss the relation between a type III TGFβ receptor, Endoglin, and Systemic Sclerosis. The TGFβ pathway, in fact, is deeply involved in SSc pathogenesis but the mechanisms of disease development are poorly understood.

This work confirmed that ENG has a pivotal role in activating different TGFβ-stimulated pathways that can be crucial in SSc, and its measurement could be useful in both diagnosis and prognosis. In fact, high concentrations of sENG seem to be related to DU, telangiectases and cutaneous and lung fibrosis development, although these data need further confirmation. Quantification of Endoglin levels can also be a starting point for the creation of specific anti-fibrotic treatments which could be used early in SSc management, to avoid or delay the onset of the characteristic disease marks.

As already suggested, additional studies highlighting ENG involvement in SSc, both clinical (i.e., further evaluations of sENG concentrations in patients with SSc in different stages of disease, with different pathotypes and compared to other connective tissue diseases) or molecular (i.e., Short vs. Long ENG, involvement of post-transcriptional mechanisms in ENG expression regulation), can pave the way to novel diagnostic, prognostic and therapeutic approaches ameliorating patients' global health and quality of life.

## Data availability statement

The original contributions presented in the study are included in the article/supplementary material, further inquiries can be directed to the corresponding author.

## Author contributions

LC and CO: conceptualization and supervision. SG, AS, GS, BP, CC, and CN: study design. SG, AS, GS, BP, CC, CN, and CO: data analysis and writing. All authors contributed to the article and approved the submitted version.

## Funding

This work was supported by the CO Italian Ministry of Education, University and Research to the DMM University of Pavia Dipartimenti di Eccellenza 2018-2022.

## Conflict of interest

The authors declare that the research was conducted in the absence of any commercial or financial relationships that could be construed as a potential conflict of interest.

## Publisher's note

All claims expressed in this article are solely those of the authors and do not necessarily represent those of their affiliated organizations, or those of the publisher, the editors and the reviewers. Any product that may be evaluated in this article, or claim that may be made by its manufacturer, is not guaranteed or endorsed by the publisher.
